# The acute effects of release techniques applied to the plantar flexors of the foot on muscle stiffness, fascicle length, and range of motion

**DOI:** 10.1590/1806-9282.20261122

**Published:** 2026-11-09

**Authors:** Tahir Keskin, Mesut Ergan, Veysel Atilla Ayyildiz, Bayram Sonmez Unuvar, Zeliha Baskurt, Ferdi Baskurt

**Affiliations:** 1 Süleyman Demirel University, Faculty of Health Sciences, Department of Physical Therapy and Rehabilitation – Isparta, Turkiye.; 2 Kocaeli City Hospital – Kocaeli, Turkiye.; 3 KTO Karatay University, Faculty of Health Sciences, Department of Physical Therapy and Rehabilitation – Konya, Turkiye.

**Keywords:** Athletic tape, Elasticity imaging techniques, Muscle tonus, Range of motion, Static stretching

## Abstract

**OBJECTIVE:**

Flexibility of the plantar flexors is important for preventing musculoskeletal injuries and ensuring proper muscle function. Static stretching, kinesiotape, and foam rolling are commonly used to enhance muscle flexibility. The aim of this study was to evaluate the acute effects of static stretching, kinesiotape, and foam rolling on muscle stiffness, fascicle length, and range of motion.

**METHODS:**

This randomized controlled trial involved 42 physiotherapy students randomly assigned to static stretching, kinesiotape, or foam rolling. Range of motion, weight-bearing lunge test, and shear wave elastography were measured before and after the interventions.

**RESULTS:**

All three interventions significantly increased range of motion and weight-bearing lunge test and decreased Achilles tendon stiffness (p<0.01). Gastrocnemius medialis stiffness decreased only after kinesiotape (p=0.005). Soleus stiffness decreased after kinesiotape (p=0.005) and foam rolling (p=0.017). Gastrocnemius medialis fascicle length increased after static stretching (p=0.014) and foam rolling (p=0.008), and gastrocnemius lateralis fascicle length increased after kinesiotape (p=0.049) and foam rolling (p=0.045). No significant differences were found between the three treatments on direct comparison.

**CONCLUSION:**

Static stretching, kinesiotape, and foam rolling are all effective in acutely increasing range of motion and weight-bearing lunge test while reducing Achilles tendon stiffness. Each technique also has specific effects on muscle stiffness and fascicle length. No single technique was superior, so method selection can be based on ease of application, equipment availability, and patient comfort.

## INTRODUCTION

Flexibility refers to the ability of a muscle to move through an unrestricted range of motion (ROM) across one or more joints. Because skeletal muscle is the largest tissue crossing a joint, muscle flexibility and stiffness are consequential for joint mobility, and inflexibility is a predisposing factor for musculoskeletal injury, overuse injury, postural disorders, and loss of ROM^1^. The triceps surae is one of the largest and strongest muscle complex of the lower limb and is exposed to substantial tensile forces during contraction and elongation^2^, making it one of the muscles particularly prone to shortening in the lower extremity. Sufficient flexibility of this complex is therefore clinically important.

Static stretching (SS) is among the most widely used techniques to improve flexibility in clinical practice^3^, reducing the stiffness and passive torque of the muscle-tendon unit and forming part of routine rehabilitation and exercise programs. Passive alternatives include foam rolling (FR) and kinesiotape (KT). FR is commonly used before sporting activity and in rehabilitation, reducing fascial adhesions^4^ and acutely increasing ROM and flexibility depending on application time and area^5^. KT is used in musculoskeletal rehabilitation to enhance muscle strength, athletic performance, ROM, and proprioception^6,7^, although its acute effects on local muscle tone and stiffness remain inconsistent across studies.

Shear wave elastography (SWE) is an objective, non-compressive method that quantifies passive muscle stiffness, viscosity, and elasticity using acoustic radiation force^8^, and its use is increasing in musculoskeletal research^9^. Despite the common clinical use of SS, KT, and FR, no study has directly compared their acute effects on the plantar flexors using SWE. The aim of this study was therefore to determine (1) the acute change in stiffness of the gastrocnemius-Achilles tendon complex, (2) the effect of each application on muscle fascicle length, and (3) whether the three applications differ in their effects. These findings may guide technique selection in sports rehabilitation and pre-warm-up practice.

## METHODS

### Participants

This study was conducted on physiotherapy students at the Faculty of Medicine, Suleyman Demirel University. Volunteers able to follow the practitioner's instructions were included; those with a lower-extremity injury in the previous 2 years, neurological problems, or corticosteroid use were excluded. Participants were instructed to avoid unusual amounts of walking, running, or sitting for 2 weeks and strenuous exercise for 2 h before testing. Written informed consent was obtained, and the study was approved by the ethics committee of the KTO Karatay University Faculty of Medicine (decision no. 2023/035). A priori power analysis (G*Power 3.1, Heinrich Heine University, Düsseldorf, Germany; effect size=0.52, α=0.05, power=0.80) indicated 13 participants per group. Of 45 participants enrolled, 3 did not meet inclusion criteria, and the study was completed with 42 participants (Figure 1).

**Figure 1:**
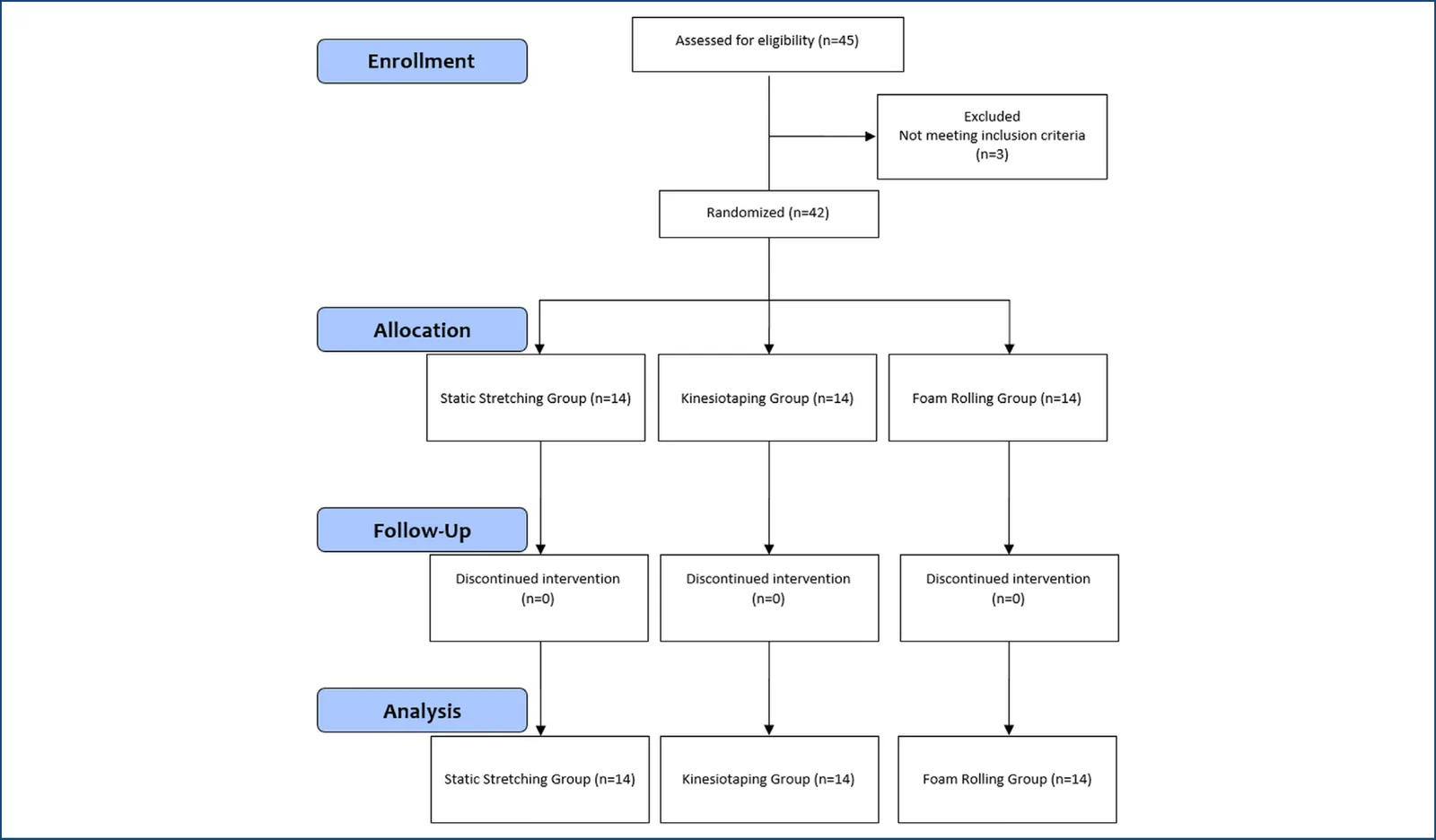
CONSORT flow diagram.

### Protocol

Participants were randomly allocated to three groups of 14 using computer-assisted block randomization (randomizer.org). SS, KT, or FR was applied to the plantar flexors of the dominant limb. ROM, the weight-bearing lunge test (WBLT), and SWE were assessed before and after the intervention by the same physiotherapist and radiologist.

### Outcome measures

Ankle dorsiflexion was measured with a universal goniometer (lateral malleolus as the pivot, aligned with the fifth metatarsal and fibula) in supine, with the participant performing maximal dorsiflexion; the mean of three trials was recorded. The WBLT was performed as a forward lunge with the heel grounded and knee touching the wall, and the toe-to-wall distance (cm) was averaged over three trials^10,11^.

SWE was performed by an experienced radiologist (Philips EPIQ-7, 18-4 MHz linear probe, 2D-SWE) with participants prone, knees extended, and the foot hanging relaxed off the table. The probe was placed longitudinally over the gastrocnemius medialis (GCM), gastrocnemius lateralis (GCL), soleus, and Achilles tendon, avoiding probe pressure that could artificially affect stiffness^12^. A color-map box was positioned in a vessel-free area; once a homogeneous confidence map was achieved, a 5-s clip was recorded and three 10-mm Regions of Interests were sampled to obtain mean stiffness (kPa). GCM and GCL fascicle lengths were measured from the same images using RadiAnt DICOM Viewer (Medixant).

### Interventions

For SS, participants maximized ankle dorsiflexion on a 30° incline board with legs vertical and held the position, relaxed, for 5 min^2^. For KT, an inhibitory Y-strip was applied prone from the calcaneus with the ankle maximally dorsiflexed and no tension along the Achilles tendon, then extended 25% over the mid-gastrocnemius with both tails anchored at the popliteal fossa^13^. For FR, a medium-density roller (Blackroll AG) was applied to the plantar flexors for three sets of 1-min proximal-distal rolling (30 cycles/min, metronome-paced) with 30-s rest between sets; participants sat with the dominant leg on the roller, the other leg crossed over it, and self-selected pressure of 7/10^14,15^.

### Statistical analysis

Data were analyzed in Statistical Package for the Social Sciences v.22.0 (IBM Corp.). Descriptive statistics are presented as mean±standard deviation. Normality was confirmed with the Kolmogorov-Smirnov test. Pre-post change within each group was assessed with paired-samples t-tests, and between-group differences with one-way analysis of variance. Significance was set at p<0.05.

## RESULTS

Sociodemographic characteristics did not differ between groups (Table 1). All three interventions produced a significant increase in ROM and WBLT and a significant decrease in Achilles tendon stiffness (p<0.01). GCM stiffness decreased significantly only after KT (p=0.005), while soleus stiffness decreased after both KT (p=0.005) and FR (p=0.017). GCM fascicle length increased significantly after SS (p=0.014) and FR (p=0.008), and GCL fascicle length increased after KT (p=0.049) and FR (p=0.045). No other within-group changes reached significance (Table 2).

**Table 1:** Sociodemographic characteristics of the participants.

Variable	Group	Mean	SD	p
Age (years)	SS	22.57	1.65	0.066^a^
	KT	22.57	1.34	
	FR	21.57	0.51	
Height (cm)	SS	1.68	0.10	0.613^a^
	KT	1.70	0.10	
	FR	1.72	0.10	
Weight (kg)	SS	67.42	21.84	0.886^a^
	KT	69.57	12.22	
	FR	66.85	9.08	
BMI (kg/m^2^)	SS	23.45	5.44	0.571^a^
	KT	23.78	3.16	
	FR	22.43	0.78	
Gender, F/M (n)	SS	8/6	-	0.683^b^
	KT	8/6	-	
	FR	6/8	-	

SS: static stretching; KT: kinesiotape; FR: foam rolling; BMI: body mass index; SD: standard deviation; F/M: female/male.

a One-way ANOVA;

b Chi-square test.

**Table 2:** Pre-post and between-group changes in range of motion, weight-bearing lunge test, muscle stiffness, and fascicle length after static stretching, kinesiotape, and foam rolling.

Variable	Group	Pre (Mean±SD)	Post (Mean±SD)	Within p	Δ (Mean±SD)	Between p
ROM (°)	SS	4.42±4.63	8.21±4.13	<0.001	3.78±1.76	0.138
	KT	12.64±5.21	16.81±5.61	<0.001	4.21±2.08	
	FR	11.21±4.42	16.71±4.53	<0.001	5.50±2.92	
WBLT (cm)	SS	10.97±1.29	12.55±1.85	<0.001	1.57±0.99	0.058
	KT	12.60±2.76	14.17±2.60	<0.001	1.57±0.72	
	FR	9.87±3.69	12.44±3.28	<0.001	2.56±1.53	
GCM stiffness (kPa)	SS	11.33±3.53	10.20±3.83	0.078	1.12±2.20	0.986
	KT	12.11±4.07	10.90±4.10	0.005	1.21±1.34	
	FR	13.76±3.50	12.51±3.28	0.081	1.25±2.48	
GCL stiffness (kPa)	SS	10.12±3.17	9.32±2.76	0.072	0.80±1.53	0.301
	KT	11.50±5.05	10.75±5.16	0.059	0.75±1.37	
	FR	11.27±2.96	9.64±1.77	0.009	1.62±1.97	
Soleus stiffness (kPa)	SS	15.66±6.48	14.20±5.18	0.215	1.46±4.19	0.632
	KT	17.78±7.35	16.06±7.34	0.005	1.72±1.90	
	FR	16.88±6.81	14.25±5.52	0.017	2.63±3.61	
Achilles tendon stiffness (kPa)	SS	43.07±12.62	35.59±11.18	0.001	7.48±6.76	0.053
	KT	63.48±18.40	48.63±18.03	<0.001	14.85±8.65	
	FR	49.02±18.80	43.82±14.65	0.005	5.20±5.86	
GCM fascicle length (mm)	SS	4.54±0.48	4.77±0.47	0.014	0.22±0.30	0.791
	KT	4.43±0.61	4.62±0.59	0.066	0.19±0.36	
	FR	4.53±0.41	4.81±0.50	0.008	0.28±0.33	
GCL fascicle length (mm)	SS	4.60±0.50	4.65±0.52	0.195	0.05±0.15	0.326
	KT	4.47±0.62	4.55±0.68	0.049	0.08±0.14	
	FR	4.88±0.40	5.05±0.52	0.045	0.16±0.28	

SS: static stretching; KT: kinesiotape; FR: foam rolling; ROM: range of motion; WBLT: weight-bearing lunge test; GCM: gastrocnemius medialis; GCL: gastrocnemius lateralis; SD: standard deviation; Δ: difference between pre- and post-intervention values, kPa: kilopascal, mm: millimeter. Within p: paired-samples t-test; Between p: one-way ANOVA on Δ values.

Between-group comparisons of pre-post change (Δ) showed no significant differences for ΔROM, ΔWBLT, or any stiffness or fascicle-length measure (all p>0.05), although ΔAchilles tendon stiffness approached significance, with KT (14.85±8.65 kPa) showing a numerically greater change than SS (7.48±6.76 kPa) and FR (5.20±5.86 kPa) (p=0.053) (Table 2).

## DISCUSSION

All three applications reduced Achilles tendon stiffness and increased ROM and WBLT. KT additionally reduced GCM stiffness, FR reduced GCL stiffness, and both KT and FR reduced soleus stiffness; GCM fascicle length increased after SS and FR, and GCL fascicle length increased after KT and FR. No application was superior to the others on direct comparison.

Adequate plantar flexor flexibility is important for daily and sporting activity and for injury prevention, and SS is the most widely used pre-activity technique, consistently shown to improve dorsiflexion ROM^16^. Previous studies report reduced GCM and GCL stiffness after SS^2,8,17^, whereas in the present study only soleus and Achilles tendon stiffness decreased significantly. This discrepancy may reflect heterogeneity in stretching duration (2–5 min across studies) and in measurement site, since passive stiffness can vary regionally within the same muscle^2^.

FR has previously been shown to acutely increase ROM to an extent similar to SS^14,18^, consistent with our findings, and the two techniques did not differ significantly here. A recent trial using myotonometry over the gastrocnemius-Achilles complex similarly found that a single FR bout significantly reduced gastrocnemius stiffness and increased ankle dorsiflexion ROM^19^, supporting the pattern observed here with SWE. In this study, FR also reduced GCL and soleus stiffness and increased fascicle length, suggesting an effect that extends beyond muscle to fascia and tendon; the outcome may also depend on application duration and number of sets, as a longer FR protocol has been shown to increase ROM more than a single 30-s bout^14,20^.

KT is thought to create space between skin and fascia and improve local circulation, an effect linked to reduced tissue edema in clinical populations^21^; quantitative myotonometric assessment of myofascial tissue mechanics has similarly been used to characterize the acute effects of KT in the lumbar region^22^. However, a placebo-controlled trial found no significant effect of KT on gastrocnemius muscle tone or extensibility^6^, and KT applied as a facilitator has been shown to increase forearm muscle stiffness, indicating that the direction of application (inhibitory vs. facilitatory) strongly influences the outcome^23^. In the present study, an inhibitory technique reduced Achilles tendon, GCM, and soleus stiffness and increased GCL fascicle length. Evidence on KT and ROM or function is similarly mixed: a systematic review in athletes with chronic ankle instability reported functional benefits of KT^24^, whereas another systematic review concluded that KT did not improve ankle function or performance compared with sham or alternative interventions^25^. Here, KT increased both goniometric ROM and WBLT.

No prior study has directly compared SS, KT, and FR for plantar flexor flexibility. Our findings indicate that all three acutely increase ROM and WBLT, reduce Achilles tendon stiffness, and increase fascicle length, without clear superiority of one technique over another; selection may therefore reasonably be guided by ease of application, equipment availability, treatment duration, and patient comfort.

Limitations include that SWE measurements were restricted to specific muscle sites and cannot be generalized to the entire muscle, and that only acute effects were assessed, so the duration of the observed changes remains unknown.

## CONCLUSION

SS, KT, and FR each significantly reduced Achilles tendon stiffness and increased ROM and WBLT, with additional application-specific reductions in muscle stiffness and increases in fascicle length. None of the three techniques was superior to the others. In sports rehabilitation, particularly for pre-warm-up stretching, the choice among these techniques can therefore be guided by ease of application, equipment requirements, time, and patient comfort.

## Data Availability

The datasets generated and/or analyzed during the current study are available from the corresponding author upon reasonable request.
